# NEIGHBORHOOD WALKABILITY AND SOCIOECONOMIC CORRELATES OF STEP ACTIVITY FOR NON-HISPANIC BLACK OLDER ADULTS

**DOI:** 10.1093/geroni/igae098.0623

**Published:** 2024-12-31

**Authors:** Kyle Moored, Michael Desjardins, Vijay Varma, Emily Richards, Sayuri Ohgi, Patrick Donahue, Michelle Carlson

**Affiliations:** Johns Hopkins Bloomberg School of Public Health, Baltimore, Maryland, United States; Johns Hopkins University, Baltimore, Maryland, United States; Clinical and Translational Neuroscience Section, National Institute on Aging, Baltimore, Maryland, United States; Johns Hopkins University, Baltimore, Maryland, United States; Johns Hopkins University, Baltimore, Maryland, United States; Johns Hopkins University, Baltimore, Maryland, United States; Johns Hopkins University, Baltimore, Maryland, United States

## Abstract

Neighborhood socioeconomic resources and walkability (e.g., proximity of services/amenities) may promote health in later life by encouraging physical activity. Yet, structural racism (e.g., redlining) has caused Black Americans to disproportionately reside in more disadvantaged neighborhoods. We examined whether neighborhood socioeconomic disadvantage, both independently and jointly with neighborhood walkability, was cross-sectionally associated with objective step activity in non-Hispanic Black older residents of Baltimore City, MD. Participants were from the Baltimore Experience Corps Trial at baseline (n=168, mean age=67.0±5.5). Neighborhood measures included validated indices of Census tract-level walkability (e.g., transit access, street connectivity, service/commercial density) and socioeconomic disadvantage (e.g., poverty, unemployment). Individuals were further categorized into combined walkability/disadvantage groups using median splits of each index: low walkability/low disadvantage (40%), high walkability/low disadvantage (7%), high walkability/high disadvantage (46%), low walkability/high disadvantage (8%). Average daily step activity was measured using an ankle-worn monitor (mean=4.3 wear days). Linear regression was used to examine associations with step activity adjusted for age, gender, and education. Being in the highest (vs. lowest) tertile of disadvantage was associated with 1513 fewer average steps per day (95% CI: -2873,-153). While walkability was not independently associated with step activity (p>.05), the high walkability/high disadvantage group had an average 1381 more steps per day (95% CI: 204,2558) compared to the low walkability/high disadvantage group. Higher neighborhood socioeconomic disadvantage was linked with lower physical activity for non-Hispanic Black older residents of Baltimore City. Yet, positive neighborhood attributes like walkability may buffer against socioeconomic disadvantage, potentially by providing access to services/amenities that promote activity.

